# Drivers of child marriage in specific settings of Ethiopia, Indonesia, Kenya, Malawi, Mozambique and Zambia – findings from the Yes I Do! baseline study

**DOI:** 10.1186/s12889-023-15697-6

**Published:** 2023-04-28

**Authors:** Maryse C. Kok, Tasneem Kakal, Abeje Berhanu Kassegne, Irwan M. Hidayana, Alister Munthali, J. Anitha Menon, Paulo Pires, Tabither Gitau, Anke van der Kwaak

**Affiliations:** 1 KIT Royal Tropical Institute, Amsterdam, the Netherlands; 2 Department of Sociology, Addis Ababa University, Addis Ababa, Ethiopia; 3 Center for Gender & Sexuality Studies, Universitas Indonesia, Depok, Indonesia; 4 Centre for Social Research, University of Malawi, Zomba, Malawi; 5 Department of Psychology, University of Zambia, Lusaka, Zambia; 6 American University of Sharjah, UAE, India; 7 Faculdade de Ciências de Saúde, Universidade Lúrio, Nampula, Mozambique; 8 Independent Consultant, Nairobi, Kenya

**Keywords:** Child marriage, Social norms, Gender, Sexuality, Teenage pregnancy

## Abstract

**Background:**

Child marriage persists in many countries and has severe impacts on health, education, economic and social status of girls. Child marriage has many interlinked causes. This study aimed to explore the drivers of child marriage in specific contexts in Ethiopia, Indonesia, Kenya, Malawi, Mozambique and Zambia.

**Methods:**

The study combined a household survey among youth (15-24 years) with focus group discussions and interviews conducted with youth (15-24 years) and parents. A variety of community stakeholders were interviewed as well. Logistic regression was done to explore associations between individual and family-level characteristics of young women and the occurrence of child marriage. Transcripts were analysed using an inductive approach. Narratives on the main drivers of child marriage across study contexts were written and inspired by the theory of normative spectrum.

**Results:**

A lack of education was associated with the occurrence of child marriage in Ethiopia, Kenya and Zambia. In all countries, teenage pregnancy was associated with child marriage. In Ethiopia, Kenya and Mozambique, fathers’ education seemed a protective factor for child marriage. Narratives of study participants showed that in Ethiopia, Indonesia and (to a lesser extent) Kenya, child marriage was perceived as an ‘appropriate practice’ to avoid premarital sex or pregnancy, whether it involved sex with or without consent. In all countries, child marriage was driven by difficult economic circumstances, which were often intertwined with disapproved social circumstances, in particular teenage pregnancy, in case of Kenya, Malawi, Mozambique and Zambia. These circumstances made child marriage an ‘acceptable practice’. Some youth, particularly in Indonesia, made their own choices to marry early, making child marriage a ‘possible practice’.

**Conclusions:**

Multiple intersecting drivers, which were present in different degrees in each country setting, influenced the occurrence of child marriage. We found that child marriage is a manifestation of social norms, particularly related to girls’ sexuality, which are intersecting with other factors at individual, social, material, and institutional level – most prominently poverty or economic constraints. Child marriage was, in some cases, a result of girls’ agentic choices. Efforts to prevent child marriage need to take these realities of girls and their families into account.

**Supplementary Information:**

The online version contains supplementary material available at 10.1186/s12889-023-15697-6.

## Introduction

Child marriage, defined as any legal or customary union involving a girl or a boy below the age of 18, is a practice that disproportionally affects girls, with negative impacts on their health, education, economic and social status [1]. Health risks associated with child marriage relate to early childbearing, where obstructed or prolonged labour can result in maternal morbidity and mortality [2, 3]. Other health effects include maternal malnutrition, isolation and depression, and sexually transmitted infections [3]. It is also well established that child marriage has negative (health) effects on children born from child mothers [4]. The negative impact of child marriage on girls’ education is profound, and because of this, young women often have less (economic) opportunities in life [1, 5]. Child marriage perpetuates gender inequality and has shown to be associated with increased intimate partner violence [6].

Evidence on the negative impacts of child marriage have, together with global, regional and national advocacy efforts and agreements, led many national governments to establish laws against the practice. While laws that set the minimum age for marriage at 18 or older have reduced child marriage prevalence in many countries [7, 8], the practice remains widespread. Globally, one in five girls get married before 18 years – with higher prevalence in parts of sub-Saharan Africa and Asia [7, 9]. In some countries, girls can legally marry with parental or judicial consent [8].

Whether legal or not, child marriage is a practice influenced by a multitude of factors. Studies show that social norms influence expectations about when a girl is supposed to get married. These include gender norms, which can impede girls’ and women’s decision-making and self-determination, prioritise boys’ education above that of girls, control girls’ sexuality^1^ and block access to information and services [1, 10]. In some settings, child marriage is seen as a tradition or is (perceived to be) recommended or mandatory by faith [11, 12]. Socio-economic factors also influence the occurrence of child marriage. Child marriage is most prevalent in poor communities, where marriage can be seen as a viable option in a context of few educational and economic opportunities [1, 12, 13]. Bride wealth can influence families to marry daughters early, and in contexts where a bride price is not common, economic relief to the family is still a driver, as financial responsibility shifts from the girls’ family to the husband [12, 13]. Family matters and family honour can also play a role in child marriage. In case of the latter, child marriage is used as a way to avoid premarital sex or getting pregnant out of wedlock [12]. Sometimes, it is argued that child marriage provides girls with security, in particular in areas where girls are perceived to be at high risk of harassment and physical or sexual assault [10]. A recent study on child marriage in sub-Saharan Africa found that (out-of-wedlock) pregnancy can also be a driver of child marriage [13]. In this context, premarital relationships often have a transactional nature, structured by gender norms (where men are supposed to provide for women) and influenced by a constrained economic environment [13]. In some cases, girls make agentic choices to marry, to address economic needs or to gain respect in the community [14, 15]. This non-exhaustive summary of potential drivers of child marriage shows that many of them are interrelated.

The drivers of child marriage vary across – and within – regions and countries [5]. Therefore, despite global evidence on what works in preventing child marriage is available [16, 17], it is important to gain a contextualized understanding of these drivers when planning interventions that aim to prevent child marriage in specific communities [13, 18]. This study aimed to explore the drivers of girl child marriage in six country contexts, in Ethiopia, Indonesia, Kenya, Malawi, Mozambique and Zambia.

## Methods

A mixed-methods baseline study was conducted in 2016, as part of the Yes I Do!^2^ programme (2016-2020), whose aim was to prevent child marriage, teenage pregnancy and female genital mutilation/ cutting (the latter only in Ethiopia, Indonesia and Kenya). This article reports on the findings related to child marriage.

### Theoretical background

Drivers of child marriage can be related to the individual (e.g. personal characteristics, beliefs, aspirations, attitudes), social (e.g., family, social networks and support), material (e.g., availability of information, education, others services and jobs), and institutional level (e.g., legal system, cultural values and beliefs, social norms) [19, 20]. There is a dynamic interplay between the drivers operating at these levels. In the literature on child marriage, social norms – the unwritten rules of governing acceptable behaviour in a society or group – are found to often intersect with drivers at other levels and are found to play a key role in perpetuating child marriage [19, 21–23]. Social norms (which include gender norms) are people’s beliefs about 1) what other people in one’s group do (descriptive norms) and 2) the extent to which other people in one’s group approve of a given action (injunctive norms) [24]. People can believe that “people in my community approve child marriage”, while at the same time people’s attitudes can be different (“I don’t personally approve child marriage”) [25]. The theory of normative spectrum suggests that social norms can have different degrees of influence on the practice of child marriage: the strongest norms can make the practice *obligatory*, strong norms can make it *appropriate*, weak norms can make it *acceptable* and the weakest norms can make it *possible* [21].

### Study contexts and methods

Both the quantitative and qualitative components of the study focused on the intersecting “pool” of drivers of child marriage. The study was conducted in specific areas in the six countries, where the Yes I Do programme was going to be implemented. These specific areas are not repeated in the rest of this article, instead, the country names are referred to. Contextual information per country setting is presented in Table 1.

**Table 1:** Contextual information per country setting

	**Ethiopia**	**Indonesia**	**Kenya**	**Malawi**	**Mozambique**	**Zambia**
Legal age of marriage in 2016	18 years, but dispensation can be granted for 16 years upon application by child or parent	16 years for women with parental permission, 19 years for men^a^	18 years	18 years, but 15 years with parental consent^b^	18 years, but 16 years with parental consent^c^	21 years, but 16 years with parental consent or lower than 16 years with judicial consent assuming the circumstances of the case are not contrary to public interest
Name of study area(s)	Amhara region: 1. Bahir Dar Zuria and 2. Kewet districts	1. West Nusa Tenggara - West Lombok district and 2. West Java - Sukabumi district	Kajiado county: 1. Kajiado West and 2. Kajiado Central^d^ sub-counties	Machinga district: 1. TA Liwonde and 2. TA Chikwewo^d^	Nampula province: 1. Mogovolas and 2. Murrupula^d^ districts	Eastern province: 1. Chadiza, 2. Katete^d^ 3. Petauke districts
Urban versus rural	Rural	Both urban and rural	Rural	Rural	Rural	Rural
Economic characteristics	Smallholder farming	Tourism, mining, farming	Pastoralist, cattle herding	Smallholder farming	Smallholder farming	Smallholder farming
Access to health and education	Health services reasonably accessible. Lack of access to secondary education.	Health services available, but less access youth in relation to SRH. Sufficient access to secondary education.	Health and educational services available but far.	Health services reasonably accessible. Lack of access to secondary education.	Limited health services accessibility. Lack of access to secondary education.	Health services reasonably accessible. Lack of access to secondary education.
Dominant ethnic group(s)	Amhara	West Lombok: Sasak Sukabumi: Sundanese	Masaai	Yao (majority), but also Chewa and Lomwe	Makua	Chewa (Chadiza and Katete) and Nsenga (Petauke)
Dominant religion(s)	Orthodox Christianity	Islam	Christianity	Islam (majority) and Christianity	Christianity (majority Catholicism) and Islam	Christianity
Dominant types of marriage	Informal and religious marriages	Formal and religious marriages	Informal and religious marriages	Informal and religious marriages	Informal and religious marriages	Informal and religious marriages

*SRH* Sexual and reproductive health, *TA* Traditional authority

^a^In 2019, this changed to 19 years for women and men in case of parental consent and 21 years without parental consent

^b^This exception was removed in 2017 by an amendment in the Constitution

^c^In 2019, this exception was removed

^d^In these (sub) districts, only quantitative and no qualitative data collection was conducted

In all country settings, a household survey, focus group discussions (FGDs) and in-depth interviews (IDIs) with young women and men (15-24 years, both married and unmarried) were conducted. In addition, FGDs and IDIs with parents or caregivers took place. Interviews were also held with stakeholders from the community, such as traditional and religious leaders, elderly women, teachers, health or social workers, representatives of community-based organizations; and key informants, such as district-level policy makers and non-governmental organization representatives (Table 2).

**Table 2:** Methods and participants

Method	Participants/ respondents	Ethiopia	Indonesia	Kenya	Malawi	Mozambique	Zambia
Survey	Young women and men 15-24 years	1,602	1,534	1,360	1,595	1,482	1,454
FGDs (average 8 participants per group)	- Girls 15-19 years - Young women 20-24 years - Young women 15-24 years - Boys 15-19 years - Young men 20-24 years - Young men 15-24 years - Parents and caregivers - Chiefs	2^a^ 1 8^b^ 5^c^ 1^d^ 1 4^e^ NA	4 4 NA 3 3 NA 2 NA	3 1 NA 1 2 NA 3 1	2 2 NA 2 2 NA 2 NA	2 2 NA 2 2 NA 4 NA	2 2 NA 2 2 NA 2 NA
IDIs	- Girls 15-19 years - Young women 20-24 years - Boys 15-19 years - Young men 20-24 years - Parents and caregivers - Grandmothers/ initiators - Traditional/ religious leaders - Teachers - Health/ social workers - CBO/ youth organization staff	8 2 7 6 2 NA 3 3 6 1	4 3 2 3 2 2 4 3 9 3	2 2 2 2 2 2 2 1 1 1	2 2 2 2 2 2 4 2 1 1	2 2 2 2 4 0 9 3 4 0	2 2 2 2 2 2 2 2 2 2
KIIs	- NGO staff - District-level government officials	1 7	3 9	1 4	3 4	0 0	5 3

*FGD* Focus group discussion, *IDI* In-depth interview, *KII* Key informant interview, *CBO* Community-based organization, *NGO* Non-governmental organization

^a^Of which one FGD included a participant aged 20

^b^Of which two were groups with young women 18-24 years

^c^Of which three FGDs included participants aged 20

^d^Of which one FGD included participants aged 18 and 19

^e^Of which two FGDs included grandmothers

### Sampling and data collection

In all study areas, a two-stage cluster household survey among young people (aged 15-24 years) was conducted. The survey focused on a variety of topics on sexual and reproductive health (SRH), including child marriage. The section on child marriage was based on a child marriage acceptability index developed by Plan International [26], which was adjusted per country context. Sample size calculations for women were based on an envisioned 10% reduction in the percentage of women (15–24 years) who have had a live birth or who were pregnant with their first child, over a 5-years’ time; with a power of 0.8, a significance of 0.05 and a design effect of 1.5. A smaller sample of men was added so that the female-male ratio was ± 75%-25%. In each randomly selected cluster, a set number of survey respondents were randomly selected at household level.

While the survey mostly focused on drivers of child marriage at individual level, other drivers and intersecting social norms were explored during the FGDs and interviews. Study participants were purposively sampled, ensuring a mix of gender, age, marital status and educational background [27]. Recruitment of study participants was facilitated by resource persons at community level.

The survey, FGD and interview tools were translated into local languages. The translation of important terms, for example concerning SRH and related attitudes and norms, was discussed and where needed revised during the training of the research teams. Researchers collected data in the local languages, considering gender and age of interviewer and participant. Pre-testing of data collection tools prompted small adjustments in each country, for example to solve problems with skipping patterns in the survey and to increase participants’ understanding of the questions. Survey data were collected with tablets, and all data collection took place in locations guaranteeing privacy. Daily feedback sessions were held among the researchers involved, to discuss key observations and refine lines of enquiry as needed.

### Data analysis

Quantitative data were analysed in Stata 15 using descriptive statistics for demographic data and data regarding respondents’ perspectives on child marriage. A regression analysis was conducted to explore the relationship between child marriage (among women 18-24 years) and various individual and family-level demographic characteristics. The choice for focusing the regression on women between 18 and 24 years was based on younger women (15-17 years) still having the possibility of marrying later, but before the age of 18. Furthermore, the age range 18-24 years provided an appropriate sample size for the regression analysis. Odds ratios are presented along with robust standard errors.

FGDs and interviews were transcribed in English and randomly checked against audio files in all countries, except in Mozambique, where summaries of the discussions were written. Qualitative data were inductively coded in NVivo by country research teams, taking into consideration the above presented theoretical background. Eight researchers (one of each country and two researchers from the KIT Royal Tropical Institute) developed summaries per main theme (on drivers of child marriage) into a matrix for cross-country analysis. Summaries per theme were written, thereby comparing outcomes of different settings and triangulating them with quantitative data where possible.

## Results

Table 3 presents selected demographic characteristics and survey outcomes for the subset of young women aged 18-24 years in all country settings, for which the regression analysis was conducted^3^. Full details on this sub-group of survey respondents and demographic characteristics of all survey respondents are presented in Additional file 1. The child marriage rate was highest in Mozambique (69%) and lowest in Zambia (14%) (Table 3).

**Table 3:** Demographic characteristics of female survey respondents (18-24 years)

	**Ethiopia**	**Indonesia**	**Kenya**	**Malawi**	**Mozambique**	**Zambia**
**Individual characteristics**						
Age	20.1 (2.0)	20.7 (1.9)	20.8 (2.02)	20.9 (1.9)	20.75 (2.0)	20.1 (2.0)
Child marriage	0.38 (0.48)	0.20 (0.40)	0.20 (0.40)	0.18 (0.38)	0.69 (0.46)	0.14 (0.35)
Some level of education^a^	0.81 (0.39)	0.99 (0.06)	0.76 (0.42)	0.96 (0.20)	0.14 (0.35)	0.93 (0.24)
Paid employment	0.45 (0.49)	0.25 (0.43)	0.81 (0.38)	0.31 (0.46)	0.88 (0.33)	0.10 (0.30)
Teenage pregnancy	0.22 (0.41)	0.29 (0.45)	0.39 (0.48)	0.55 (0.49)	0.27 (0.44)	0.38 (0.48)
Ever received any form of SRH information/ education	0.79 (0.40)	0.77 (0.42)	0.86 (0.34)	0.82 (0.38)	0.52 (0.49)	0.76 (0.42)
**Family-level characteristics**						
*Parental education (some level of education*^*a*^*)*						
Mothers	0.08 (0.27)	0.85 (0.36)	0.21 (0.40)	0.40 (0.49)	0.59 (0.49)	0.47 (0.49)
Fathers	0.17 (0.37)	0.88 (0.32)	0.22(0.41)	0.62 (0.48)	0.47 (0.49)	0.63 (0.47)
*Household size*						
One to two	0.28 (0.44)	0.54 (0.22)	0.05 (0.21)	0.09 (0.29)	0.09 (0.29)	0.03 (0.19)
Three to four	0.34 (0.47)	0.42 (0.49)	0.30 (0.45)	0.48 (0.50)	0.44 (0.49)	0.23 (0.42)
Five to seven	0.30 (0.46)	0.44 (0.49)	0.41 (0.49)	0.31 (0.46)	0.37 (0.48)	0.35 (0.47)
Eight or more	0.08 (0.27)	0.75 (0.26)	0.23 (0.42)	0.11 (0.31)	0.081 (0.27)	0.37 (0.48)
**Total observations**	682	611	474	619	602	335

Data are presented in means (standard deviation), the last row presents numbers

^a^This included: mainstream primary/ secondary education, vocational training, or madrassa depending on the country

Table 4 contains the regression outcomes regarding drivers of child marriage among young women aged 18-24 years. In Ethiopia, Malawi and Mozambique, older respondents (within the age range of 18-24 years) were less likely to have experienced child marriage than younger respondents. Having no education was associated with higher likelihood of child marriage in Ethiopia, Kenya and Zambia. Having ever dropped out of school was associated with child marriage in Indonesia. Respondents’ paid employment did not yield significant associations, except in Malawi, where it was found to be associated with child marriage. In all countries, teenage pregnancy was associated with child marriage. Having ever received any form of SRH information or education showed mixed associations with child marriage across the countries. In relation to family-level characteristics, fathers’ education seemed a protective factor for child marriage in Ethiopia, Kenya and Mozambique. The data on household size show that respondents living alone or with another person were more likely to have experienced child marriage in Indonesia and Zambia compared to those living with 5-7 persons.

**Table 4:** Regression results on drivers of child marriage among female survey respondents (18-24 years)

	**Ethiopia**	**Indonesia**	**Kenya**	**Malawi**	**Mozambique**	**Zambia**
**Individual characteristics**						
Age	0.841** (0.0446)	0.887 (0.0619)	1.066 (0.0948)	0.812** (0.0549)	0.873** (0.0415)	0.928 (0.0944)
Some level of education	0.526* (0.138)	NA	0.153** (0.0540)	0.446 (0.228)	1.069 (0.312)	0.259* (0.164)
Having ever dropped out of school^a^	NA	2.175* (0.772)	NA	NA	NA	NA
Paid employment	0.731 (0.169)	0.762 (0.282)	0.554 (0.288)	1.870* (0.456)	1.279 (0.387)	0.836 (0.504)
Teenage pregnancy	10.56** (3.029)	30.91** (9.335)	27.92** (13.97)	17.44** (7.073)	6.374** (1.785)	19.77** (10.22)
Ever received any form of SRH information/ education	9.395** (4.176)	0.396** (0.130)	2.170 (1.353)	2.059 (0.759)	0.707 (0.137)	0.670 (0.301)
**Family-level characteristics**						
Mother having some level of education	0.906 (0.312)	0.275** (0.136)	0.793 (0.476)	0.895 (0.246)	1.156 (0.276)	1.054 (0.455)
Father having some level of education	0.542* (0.147)	1.803 (0.955)	0.364* (0.185)	1.558 (0.418)	0.614* (0.145)	1.526 (0.691)
Household size: one to two	1.606 (0.411)	4.742** (2.486)	NA^b^	0.807 (0.484)	1.038 (0.398)	9.980* (11.19)
Household size: three to four	1.435 (0.359)	1.079 (0.328)	1.047 (0.368)	1.321 (0.372)	1.029 (0.211)	1.764 (0.791)
Household size: five to seven^c^	NA	NA	NA	NA	NA	NA
Household size: eight or more	0.839 (0.327)	0.926 (0.409)	0.442 (0.197)	0.282* (0.177)	0.636 (0.255)	0.668 (0.334)
**Other characteristics**						
Study area 1	0.741 (0.171)	0.971 (0.318)	2.766** (0.930)	0.733 (0.177)	0.781 (0.153)	0.498 (0.251)
Study area 3 (with study area 2 as reference)	NA	NA	NA	NA	NA	1.333 (0.666)
Constant	3.166 (3.357)	1.422 (2.158)	0.00926* (0.0187)	1.680 (2.586)	2.539 (2.835)	0.355 (0.756)
Total observations	682	611	474	619	602	335

Data are presented on odds ratio (standard error), significant levels are indicated with ** *p<*0.01, * *p<*0.05

The regression model was focused on drivers of child marriage, however, some of those could also be consequences of child marriage

^a^As in Indonesia there were no big differences in education in the sample, having ever dropped out of school was included in the regression

^b^In Kenya, only 24 respondents indicated to be in a household of one or two people, therefore, this dropped out of the analysis

^c^With regard to household size, the reference category was a household size of 5-7 persons

The quantitative and qualitative data revealed a multitude of drivers of child marriage in the six countries, which are summarized in Table 5 per level (individual, social, material and institutional). Below, we present the intersections of the main drivers across these levels, with particular attention to underlying social norms, in three overarching narratives: 1) child marriage as ‘appropriate practice’ to avoid or protect girls from premarital sex; 2) child marriage as ‘acceptable practice’ when there are limited perceived (economic and social) alternatives; and 3) child marriage as ‘possible practice’ in case of girls’ agentic choice.

**Table 5:** Summary of identified drivers of child marriage in the six country contexts

**Level**	**Drivers of child marriage**	**Observed in country context**
Individual	Lack of education^a^ School drop-out^a^ Teenage pregnancy Agentic choice to marry early Lack of knowledge about legal age of marriage	Ethiopia, Kenya, Zambia Indonesia Ethiopia, Indonesia, Kenya, Malawi, Mozambique, Zambia Ethiopia, Indonesia All countries, particularly Indonesia, Kenya, Mozambique, Zambia
Social	Mothers’ lack of education^a^ Fathers’ lack of education^a^ Family honour, status Family bonds, relationships Sexual relationships	Indonesia Ethiopia, Kenya, Mozambique Ethiopia, Kenya Ethiopia Ethiopia, Indonesia, Kenya, Malawi, Mozambique, Zambia
Material	Lack of job opportunities Financial constraints in girls’ family Bride wealth	Ethiopia, Indonesia, Kenya, Malawi, Mozambique, Zambia Ethiopia, Indonesia, Kenya, Malawi, Mozambique, Zambia Kenya and less in Indonesia, Zambia
Institutional	Social and gender norms	
	- Taboo of premarital sex and girls’ sexuality; importance of virginity, particularly for unmarried women; family shame in case of pregnancy out of wedlock	All countries, particularly Ethiopia and Indonesia
	- Female’s education less important than men’s education	Ethiopia, Indonesia and Kenya
	- Men to financially provide for women	All countries, particularly Malawi, Mozambique and Zambia
	- Little decision-making space for girls	All countries, particularly Ethiopia, Indonesia and Mozambique
	- Importance of transition to adulthood; unmarried women less respected than married women	All countries, particularly Ethiopia and Indonesia
	- Wives to be obedient to husbands – younger wives more obedient	All countries, particularly Ethiopia and Indonesia
	Abduction	Indonesia
	Religion	Indonesia, Ethiopia

^a^This is based on quantitative results (Table 4)

### The taboo around girls’ sexuality – child marriage as an ‘appropriate practice’

In all countries and particularly in Ethiopia, Indonesia and (to a lesser extent) Kenya, qualitative data revealed that child marriage was strongly linked to a taboo around adolescent girls’ sexuality and premarital sex. Maintaining girls’ virginity until marriage was the norm, which was attached to family honour and reputation in Ethiopia and Indonesia (see also Table 6). In Kenya, a few participants indicated that girls were expected to take responsibility in avoiding sex, while such expectations were not explicit for boys or young men – regarding their own virginity or the girl’s. For example, girls were supposed to dress properly to avoid provoking boys and young men. Similarly, in Ethiopia and Indonesia, girls were not allowed to have sex before marriage, while for boys this was less strict. Child marriage was deemed appropriate to avoid premarital sex or pregnancy for girls, whether it involved consensual sexual relationships or (fears of) sexual violence.

*“Courtship is now related to sexuality – having sex, abortion. I once worked with a student in grade 11 who was married by her parents because of concern for her social relations. They did not want to take the risk of pregnancy.”* (IDI, male high school teacher, Sukabumi district, Indonesia)

**Table 6:** Respondents' perspectives on child marriage

	**Ethiopia**		**Indonesia**		**Kenya**		**Malawi**		**Mozambique**		**Zambia**	
**Perspectives of young women (18-24 years) who married as children (% (n))**												
It was their choice to get married	30% (260)		85.7% (140)		30.5% (105)		67.1% (167)		68.8% (237)		57.5% (73)	
Felt pressured into marriage by any person or family	56.5 (260)		9.3% (140)		52.4% (105)		10.8% (167)		35.9% (237)		37.0% (73)	
**Respondents (15-24 years) who slightly agreed, agreed or strongly agreed**^**a **^**with the following statements:**												
	Females (*n=*1,127)	Males (*n=*469)	Females (*n=*1,157)	Males (*n=*377)	Females (*n=*1,019)	Males (*n=*349)	Females (*n=*1,194)	Males (*n=*401)	Females (*n=*1,222)	Males (*n=*248)	Females (*n=*1,001)	Males (*n=*447)
Marrying girls at a young age is part of our religious practices	14.6%	18.1%	56.8%	54.4%	5.8%	7.7%	7.9%	6.0%	12.9%	10.9%	5.8%	3.6%
Marrying girls at a young age can help protect family honour/ reputation	48.3%	48.0%	45.2%	52.8%	4.6%	5.4%	8.6%	6.0%	25.3%	14.1%	19.7%	10.7%
Marriage of girls under 18 years may happen because of pregnancy	36.0%	56.1%	8.6%	14.1%	75.6%	73.6%	69.0%	82.3%	54.7%	56.9%	80.0%	74.7%
Marriage of girls under 18 years mostly happens because there is a lack of education and job opportunities	43.8%	47.3%	29.6%	27.3%	73.2%	74.2%	65.7%	70.6%	46.2%	56.5%	70.8%	79.6%
Marriage of girls under 18 years sometimes happens for financial reasons	48.5%	69.1%	48.4%	51.2%	51.0%	53.6%	68.8%	71.8%	20.8%	23.8%	70.1%	61.3%
Marrying girls young can help prevent sexual violence, assault and harassment	40.1%	56.9%	39.2%	47.2%	7.2%	5.7%	13.9%	7.2%	23.0%	23.8%	18.4%	13.2%
Marrying a girl young is preferable because younger brides are more obedient and respectful of their husbands	52.6%	42.2%	55.1%	44.6%	21.2%	17.2%	27.5%	23.4%	34.9%	41.1%	34.4%	23.5%
Even if a girl does not want to be married she should honour the decisions/ wishes of her family	52.4%	56.1%	66.7%	64.2%	42.0%	42.7%	27.9%	27.2%	39.2%	39.5%	31.8%	28.9%
**Respondents’ (15-24 years) knowledge about the minimum age of marriage for girls** (Ethiopia: 18 years, Indonesia: 16 years, Kenya: 18 years, Malawi: 18 years, Mozambique: 18 years, Zambia: 21 years)												
Respondents who knew the minimum age for marriage according to statutory law for girls	74.1%	61.8%	0.9%	0.8%	21.1%	24.4%	43.0%	45.4%	16.9%	32.3%	1.9%	0.4%

^a^The answer options were: strongly disagree – disagree – slightly disagree – neutral – slightly agree – agree – strongly agree – don’t know – no answer

An older male community member (IDI) in Ethiopia, when asked about the reasons for child marriage occurring in his community, said:

*"Daughters are prone to sexual harassment or even rape if they sit outside the home and may even end up giving birth to an illegitimate child. But sons are free from these risks and hence their outside exposure may not pose as much risk for the family."*

Many young survey respondents (15-24 years) in Ethiopia and Indonesia also agreed or strongly agreed that marrying girls young can help prevent sexual violence, assault and harassment. Male respondents had higher levels of agreement than female respondents in both countries. (Ethiopia: 40.1% and 56.9% and Indonesia: 39.2% and 47.2% for female and male respondents respectively, Table 6).

In Indonesia and to a lesser extent in Ethiopia, both qualitative and quantitative data (Table 6) revealed that religion provided a justification for child marriage, to prevent premarital sex. Qualitative data revealed that in Ethiopia and Kenya, marriage and weddings were seen, more than in the other countries, as important family and community affairs. In Ethiopia, child marriage could “cement relationships between families”. In both Ethiopia and Kenya, having a daughter married meant increased prestige and respect for her family. Fathers, together with other family members, made the decisions about child marriage (corroborating with the regression finding about fathers’ education being a protective factor in half of the countries). Girls had very little space to directly communicate with the father and refuse the marriage. Girls who refused marriage were seen as undisciplined and in some cases as outcasts, where family or community pressure made the marriage almost ‘obligatory’.

*“In our community girls cannot reject a marriage proposal. Forced marriage is practiced to strengthen the family bond. Pressure to marry is on the girl, not on the boy. If a girl is over the age of 15, she has no value in the community."* (IDI, 40-year-old married female teacher, Ethiopia)

*“In most cases girls do not have a choice in this matter. It is the girl’s parents who decide, girls who are married off are not happy, they lose hope with their lives. When a girl refuses to be married off, the parent will throw a curse at her and this affects her life.”* (IDI, male religious leader, Kenya)

Quantitative data confirmed that family pressure was a prominent driver in Ethiopia and Kenya, where 56.5% of the women (18-24 years) who experienced child marriage in Ethiopia and 52.4% of them in Kenya reported that they felt pressurized into marriage by their family or any other person (Table 6).

In Ethiopia, FGD and interview participants indicated that female genital mutilation/ cutting (FGM/C) – practiced soon after birth – increases marriageability, while this connection was not found in Indonesia. In Ethiopia, community stakeholders reported that FGM/C was believed to reduce girls’ sexual desire, stimulate them to be good wives and increase future husbands' sexual pleasure. In Kenya, where FGM/C is conducted at a later age, community stakeholders indicated that having undergone FGM/C allows girls to engage in sex and makes them ready for marriage.

### Economic and social circumstances, particularly teenage pregnancy – child marriage as an ‘acceptable practice’

In all countries, child marriage was a response to difficult economic circumstances, which were often intertwined with disapproved social circumstances, in particular teenage pregnancy, in the case of Kenya, Malawi, Mozambique and Zambia.

In all countries, many young and older participants explained that child marriage was prevalent because it reduces financial burden on the girls’ family. Survey respondents also indicated that marriage of girls under the age of 18 sometimes happens for financial reasons (Table 6).

*“The problem is as follows... The father is the head, he tells her to get married. This helps to limit buying clothes and food for the girl. There are many people who do this.”* (IDI, community leader, Mozambique)

A few participants in Kenya also spoke about pre-arranged marriages that were related to finances. In particular in Kenya, and in some cases in Ethiopia, Indonesia and Zambia, the bride price motivated families to marry off their daughters, to be able to ease financial problems or pay debts.

[On the benefits of child marriage] *“Not really. But the parents will be relieved of the burden of educating her [teenager mother] as well as taking care of her child. The parents will also benefit from the dowry they will receive.”* (IDI, 23-year-old married woman, Kenya)

*“Children of 13, 14, 15 are in marriages and mostly are forced by their parents because they want to make money out of it.”* (FDG, young women 15-19 years, Zambia)

In Malawi, Mozambique and Zambia, many FGD and interview participants spoke about the difficulties for young people to continue studying and find jobs, especially for girls and young women. This also emerged from the quantitative data, where many respondents from these three countries, including Kenya, agreed or strongly agreed that child marriage mostly happens because there is a lack of education and job opportunities (Table 6). Young people in FGDs and interviews said that they felt there were little prospects and thus alternatives to marriage. While in these countries, participants mainly referred to difficult economic circumstances underlying this, the norm of men being responsible to (financially) provide for women also influenced the occurrence of child marriage. As one married 18-year-old young man in Mozambique put it:

*“Here in this region, when a person has a machamba [piece of land for subsistence farming] he can marry.”* (IDI, 18-year-old man, married when 17 years old, Mozambique)

The norm of men being responsible to provide for women led parents and girls to select or support the selection of potential husbands who would be able to financially provide for the girl.

*“Like the number one thing, the girls in this community: when you go to Johannesburg and come back [with money] the girls want to marry you right away. And when you go to their parents, they do not hesitate to give away their daughter.”* (IDI, 22-year-old married man, Malawi)

In Ethiopia, Indonesia and Kenya, more participants explicitly indicated that girls’ education and personal development were often considered less important than those of boys.

*“For me, women do not need a high school education, so they do not to have to work and can take care of the household.”* (IDI, 22-year-old unmarried man, Sukabumi district, Indonesia)

Above we presented that teenage pregnancy was associated with child marriage in all six countries. We also mentioned premarital teenage pregnancy as a threat to the reputation of the girls’ family and linked to this, child marriage being used as a means to avoid this. In Kenya, Malawi, Mozambique and Zambia, participants in FGDs and interviews and survey respondents (Table 6) indicated that teenage pregnancy was a primary reason for child marriage^4^. Here, child marriage was an almost automatic response to premarital pregnancy, meant to avoid further stigma and shame of the girls’ family (in Zambia, this was referred to as ‘damage’). The marriage would be arranged between the two families, however in Kenya, a teenage pregnant girl could also be married off to be the second or third wife of another, often older man, who was not responsible for the pregnancy. In Mozambique, traditional and religious leaders were said to facilitate child marriage in the case of teenage pregnancy. In this case, refusal of marriage was said to be difficult, more so in Kenya and Mozambique than in Malawi and Zambia.

*“The thing is, once they are pregnant, most of them get married, but it is not their intention to get married, the parents just say they should get married.”* (FGD, young men 15-19 years, Malawi)

In Kenya, Malawi, Mozambique and Zambia, teenage pregnancy was prevalent and very often a result of transactional sexual relationships between girls and young men. In an economically precarious context, girls would engage in sexual activity, often unprotected, with young men in exchange for money to fulfil basic needs, school fees, or for products such as lotions and dresses. In some cases, young women were involved in relationships with teachers who promised good grades.

*“The teachers here abuse the girls a lot, we had a problem with a former school director who had to leave as he made four students pregnant… but there are many teachers who make children in the area they work in, they come to work but then they marry the students.”* (IDI community leader, Mozambique)

Similar to FGM/C marking a transition to adulthood for Kenyan girls (described above), in Malawi, Mozambique and Zambia, many participants spoke about the influence of initiation ceremonies on sexual behaviour of young adolescents. Initiated boys and girls were motivated to experiment with sex, which was often unprotected. This led to teenage pregnancy and consequently, in some cases, child marriage.

### Agentic choice – child marriage as a ‘possible practice’

In all countries, the majority of the participants thought that child marriage has little benefits. However, the above narratives show that social (including gender) norms, which were often intersecting with economic and social circumstances, perpetuated the practice. Parents often made marriage-related decisions, with passive consent of the girl who was to marry. Acknowledging that social and gender norms and other contextual factors have a bearing on individuals’ choices, we also found examples of more agentic choices to marry early, particularly in Indonesia. In West Lombok in Indonesia, some participants talked about abduction: a boy or young man could take his girlfriend away from her family’s house (with or without her consent). After this, they married, as they were in love or (were perceived to have) had sexual intercourse. Sometimes, the young couple married without parental consent.

*“I begged my daughter not to marry. I wanted her to finish her education, especially as from my 13 sons and daughters, only she could study at the vocational school. But my daughter still wanted to get married, especially she had been abducted by her boyfriend. Finally she dropped out of school when she was in the second year.”* (FGD, male parents, West Lombok district, Indonesia)

According to various young and older participants in Indonesia, dating (with or without sex) was common among young people. Social media often played a role in linking young people, without the knowledge of their parents. Turning such relationships into marriage was sometimes based on the wish of the young couple to start sexual activity and avoid premarital sex (*zinah*). Such marriage could be dissolved relatively easily: if the ‘love was gone’, a divorce was settled for, as per the rapport of an elderly woman in West Lombok. Girls’ choices to marry were sometimes influenced by the fear of being left by their boyfriends if they refused to marry, or the fear that another man would ask them to marry or abduct them. It was also influenced by the (social) perception that marrying at an older age, or no marriage at all, was not good.

*“The ideal age for marriage is 18-20. For women, it’s the early 20s and for males 22. When females reach the age of 25 years, they will be regarded as mosot (spinsters). They would be mocked here… There are never unmarried people in the 30s. These people are called mosot. Those are people who nobody wants to marry.”* (IDI, male religious official, West Lombok district, Indonesia)

This also came across in some narratives from Ethiopia, where community stakeholders reported that there is no more humiliating experience for a woman than to be labelled as *kumo ker* (unable to find a husband) as this carries the social stigma of being unwanted by men.

In Mozambique, similar to Indonesia, some young men in an FGD also shared that they chose to marry as it provided them the opportunity to have sex. One married girl in Mozambique indicated to have chosen to marry herself, because her parents did not buy her clothes. In Malawi and Zambia, there were accounts of girls opting themselves for marriage to lessen financial burden on their families. In Malawi, girls in an FGD (15-19 years) seemed to long for adulthood:

*“Yeah, I have these breasts like this, I can marry and I should be cooking for myself.”* (FGD with young women 15-19, Malawi)

Married women were perceived to be adults and gained more respect in the community. In Ethiopia and Indonesia, some participants said that unmarried women had no value in the community. Peer pressure as a contributor to child marriage was mentioned by participants in Malawi and Kenya.

## Discussion

The study found multiple intersecting drivers of child marriage, which were present in different degrees in each study setting. Recently, Psaki et al. (2021) presented a framework on drivers of child marriage. Our study findings confirm that social norms or attitudes, and poverty and economic factors – in Psaki et al.’s framework presented as broader environmental factors – influence girls’ agency and opportunities, and relate to a fear of girls’ sexuality and pregnancy, which all contribute to child marriage [18]. In the six study settings, child marriage is not a norm itself, but a manifestation of social norms that are intersecting with other factors at individual, social, material, and institutional level [28, 29]. Our findings demonstrate that depending on the setting, the mix of drivers can make child marriage an appropriate, acceptable or possible practice [21]. In line with the theory of normative spectrum, strong social norms concerning the importance of maintaining girls’ virginity can make child marriage an appropriate practice. Economic and social circumstances (combined with social norms) can make child marriage an acceptable practice, and child marriage as possible practice involved the least strong (or weak) social norms, providing more space for agentic choice. Depending on the setting, these perceptions of child marriage being an appropriate, acceptable or possible practice, can exist next to each other.

The social norms connected with child marriage covered a range of domains, which is in line with existing evidence [28, 29]. A taboo on girls’ sexuality and premarital sex, sometimes but often not related to religion, led to parents marrying off their daughters to protect them from pregnancy out of wedlock in some cases, while in other cases, it translated into marriage automatically following premarital pregnancy, as found in other studies as well [12, 13, 18, 30–33]. Norms that devalue girls and women as contributors to society and economy were persistent in all settings as well. For example, girls’ education and employment was found to be less important, some girls were seen as a financial burden to the family, and their decision-making power was more constrained than that of young men. This contributed to the occurrence of child marriage [29]. Our findings also corroborate with the notion that in various settings, marriage is perceived to be central to women’s life, it provides them a way to ‘adulthood’ and community respect which, in particular in the absence of alternative economic and social opportunities, can contribute to (choosing) early marriage [34–36].

Much of this normative underpinning of (child) marriage is patriarchal and rooted in gender inequality [28, 37]. Gender norms relate to the management of girls’ sexuality and are central in shaping inequalities in power relationships and distribution of resources that, in turn, perpetuate child marriage [29, 37, 38]. Not conforming to these norms can damage families’ reputations, stigmatize unmarried girls and young women or promote their violent punishment [37].

While our study findings did not particularly focus on potential consequences of not conforming to social norms and disobedience of elders’ expectations regarding marriage, fear for sanctions was there in Ethiopia and Kenya, more than in the other countries. Religion was mentioned in Indonesia, but no in-depth analysis of its influence was possible. Other studies stress the influence of religion on child marriage in certain contexts, mostly in relation to the taboo on sex and pregnancy out of wedlock. They also point at marriage being perceived as promoting spiritual maturity in Islam, and confirming women’s subordination to men [11, 12].

In all countries, but most prominently Kenya, Malawi and Zambia, child marriage was related to gaining economic security. The Covid-19 pandemic, not elaborated in our study, has influenced educational and economic opportunities, as well as access to health services and the occurrence of teenage pregnancy, and has further increased child marriage rates in many countries [39, 40]. Conflict can have the same effect. A meta-ethnography by Kohno et al. (2020) shows that living in conflict or displacement could strengthen child marriage being perceived as ‘appropriate practice’, to protect unmarried girls from being violated and losing their virginity; or ‘acceptable practice’, to ease families’ financial pressure, or ‘possible practice’, as reaction to ‘loneliness’ [12].

Child marriage as a ‘possible practice’ has gained attention over the past years. Several scholars have pointed to the need for more respect for girls’ agentic choices for marriage within constrained contexts [36, 41–43]. Horii (2020) argues that child marriage has become ‘deviant behaviour’ and a human rights violation after the rise of modernity in the Western world, where children are regarded as innocent, marriage has become optional and based on romantic love and individual choices, and tradition is regarded as something harmful. She states that the child marriage discourse ignores the possibility of children exercising agency to marry early [42]. Indeed, in Indonesia and several other settings in this study we saw girls making these agentic choices. Realities in which these choices are made, for example ‘resolving’ problems of premarital pregnancy or economic hardship, need to be well understood to identify alternative options for the securities girls (and their families) may seek [41]. In addition, consequences of not marrying or cancelling marriages need to be considered [44].

It is clear that programmes aiming to prevent child marriage, such as Yes I Do!, need to make use of multiple, context-specific approaches that take into account all intersecting – including structural – drivers of child marriage. The Yes I Do! programme focused on intergenerational and gender transformative approaches in communities; involving girls, boys, young women and men, and community gatekeepers to challenge gender norms and roles and take action to prevent child marriage. It focused on meaningful youth participation, comprehensive sexuality education and the provision of SRH services, including contraceptives, and supporting young women and their environments in education and economic empowerment. Lastly, it aimed to enhance the implementation of laws and policies around child marriage and young people’s sexual and reproductive health and rights. Several reviews provide insights into the effect of such interventions on the occurrence of child marriage [16, 17, 45], but the effective ‘mix’ of such interventions will be context specific. For example, in Indonesia, the programme did not focus on promoting girls’ education as much as in the other countries, because most young people were in school or educated. In Southern Africa, providing access to contraceptives for youth was a focus area, while in Indonesia providing contraceptives for youth was banned. One learning from this study is the importance of programme staff discussing and enhancing understanding about the complexity of drivers of child marriage, including underlying social norms around sexuality, and participating in value clarification exercises in this regard.

The strength of this study lies in the inclusion of multiple countries and methods. It is important to repeat the note that identified drivers of child marriage cannot be generalized within and between countries. Our quantitative analysis faced limitations as there were not enough data on socio-economic status of households, and, as indicated, there are endogenous relationships between for example child marriage and education or employment. Our study did not focus on the enforcement of legal frameworks, while they influence occurrence of child marriage [12]. The qualitative component provided in-depth insights into social norms underlying child marriage, but the quantitative data only provided some directions in this regard: ‘measuring’ social norms and studying how they potentially change over time is challenging [23, 28, 46].

## Conclusions

Multiple intersecting drivers, which were present in different degrees in each country setting, influenced the occurrence of child marriage. In all settings, child marriage was a manifestation of social norms that are intersecting with other factors at individual, social, material, and institutional level – the most prominent ones being poverty or economic constraints. We found that child marriage has an important relationship with (perceptions around) sexuality, particularly of young women. Depending on the setting, the mix of drivers can make child marriage an appropriate, acceptable or possible practice. Efforts to prevent child marriage need to take these realities of girls and their families into account.

## Supplementary Information

**Additional file 1:** Demographic characteristics of the sample (regression, women 18-24 years; and full sample, female and male respondents 15-24 years).

## Data Availability

The research protocol and the datasets used and analysed during the current study are available from the corresponding author on reasonable request.

## Abbreviations

FGD: focus group discussion
FGM/C: female genital mutilation/cutting
IDI: in-depth interview
SRH: sexual and reproductive health

## Footnotes

1 Sexuality is “a central aspect of being human throughout life and it encompasses sex, gender identities and roles, sexual orientation, eroticism, pleasure, intimacy and reproduction. Sexuality is experienced and expressed in thoughts, fantasies, desires, beliefs, attitudes, values, behaviours, practices, roles and relationships. While sexuality can include all of these dimensions, not all of them are always experienced or expressed. Sexuality is influenced by the interaction of biological, psychological, social, economic, political, cultural, legal, historical, religious and spiritual factors” (WHO 2006. Defining sexual health: report of a technical consultation on sexual health, 28–31 January 2002, Geneva. Geneva: World Health Organization).

2 Yes I Do! refers to young people being able to speak up about if, when and with whom to marry and have children.

3 The total sample size for women (18-24 years) was larger in each country, however, due to missing data for some of the characteristics involved, in particular parents’ education, the sample size decreased.

4 56.1% of the male respondents (15-24 years) in Ethiopia also indicated that marriage of girls under 18 years may happen because of pregnancy (Table 6).
