# Supplementary material for: Drivers of child marriage in specific settings of Ethiopia, Indonesia, Kenya, Malawi, Mozambique and Zambia – findings from the Yes I Do! baseline study
Source: BMC Public Health. 2023 Apr 28;23:794. doi: 10.1186/s12889-023-15697-6 (PMC10141833; doi:10.1186/s12889-023-15697-6)
Supplement: Supplementary file 1 — Additional file 1: Demographic characteristics of the sample (regression, women 18-24 years; and full sample, female and male respondents 15-24 years). [file 12889_2023_15697_MOESM1_ESM.docx]

**Additional file 1**

**Demographic characteristics of the sample (regression, women 18-24 years; and full sample, female and male respondents 15-24 years)**

|  | **Ethiopia** | | **Indonesia** | | **Kenya** | | **Malawi** | | **Mozambique** | | **Zambia** | |
| --- | --- | --- | --- | --- | --- | --- | --- | --- | --- | --- | --- | --- |
|  | Women (18-24 years) | All respondents (15-24 years) | Women (18-24 years) | All respondents (15-24 years) | Women (18-24 years) | All respondents (15-24 years) | Women (18-24 years) | All respondents (15-24 years) | Women (18-24 years) | All respondents (15-24 years) | Women (18-24 years) | All respondents (15-24 years) |
| **Age** | 20.1 (2.0) | 18.6 (2.7) | 20.7 (1.9) | 18.7 (2.8) | 20.8 (2.02) | 18.4 (2.9) | 20.9 (1.9) | 19.4 (2.9) | 20.8 (2.0) | 18.8 (2.9) | 20.1 (2.0) | 18.5 (2.7) |
| Missing observations | 0 | 0 | 0 | 0 | 0 | 0 | 0 | 0 | 0 | 0 | 0 | 0 |
| **Child marriage** | | | | | | | | | | | | |
| Yes | 0.38 (0.48) | NA | 0.20 (0.40) | NA | 0.20 (0.40) | NA | 0.18 (0.38) | NA | 0.69 (0.46) | NA | 0.14 (0.35) | NA |
| No | 0.62 (0.48) | NA | 0.80 (0.40) | NA | 0.80 (0.40) | NA | 0.82 (0.38) | NA | 0.31 (0.46) | NA | 0.86 (0.35) | NA |
| Missing observations | 0 | NA | 0 | NA | 0 | NA | 0 | NA | 0 | NA | 0 | NA |
| **Having some level of education** | | | | | | | | | | | | |
| Yes | 0.81 (0.39) | 0.87 (0.33) | 0.99 (0.06) | 0.99 (0.06) | 0.76 (0.42) | 0.84 (0.35) | 0.96 (0.20) | 0.96 (0.20) | 0.86 (0.35) | 0.86 (0.34) | 0.93 (0.24) | 0.90 (0.29) |
| No | 0.19 (0.39) | 0.13  (0.33) | 0.004 (0.06) | 0.004  (0.06) | 0.23 (0.42) | 0.16 (0.35) | 0.04 (0.20) | 0.04 (0.20) | 0.14 (0.35) | 0.14 (0.34) | 0.065 (0.24) | 0.10 (0.29) |
| Missing observations | 0 | 0 | 0 | 0 | 0 | 1 | 0 | 0 | 0 | 0 | 0 | 0 |
| **Having ever dropped out of school** | | | | | | | | | | | | |
| Yes | 0.69 (0.46) | 0.52 (0.49) | 0.19 (0.39) | 0.15 (0.36) | 0.34 (0.47) | 0.22 (0.41) | 0.84 (0.37) | 0.69 (0.46) | 0.79 (0.41) | 0.67 (0.46) | 0.65 (0.47) | 0.51 (0.50) |
| No | 0.31 (0.46) | 0.48 (0.49) | 0.80 (0.39) | 0.85 (0.36) | 0.66 (0.47) | 0.78 (0.41) | 0.16 (0.37) | 0.31 (0.46) | 0.21 (0.41) | 0.33 (0.46) | 0.35 (0.47) | 0.49 (0.50) |
| Missing observations | 7 | 14 | 0 | 4 | 1 | 2 | 1 | 1 | 13 | 25 | 6 | 7 |
| **Paid employment** | | | | | | | | | | | | |
| Yes | 0.45 (0.49) | 0.36 (0.48) | 0.25 (0.43) | 0.23 (0.41) | 0.81 (0.38) | 0.12 (0.32) | 0.31 (0.46) | 0.30 (0.45) | 0.12 (0.33) | 0.10 (0.29) | 0.10 (0.30) | 0.08 (0.28) |
| No | 0.55 (0.49) | 0.64 (0.48) | 0.75 (0.43) | 0.77 (0.41) | 0.19 (0.38) | 0.88 (0.32) | 0.69 (0.46) | 0.70 (0.45) | 0.88 (0.33) | 0.90 (0.29) | 0.90 (0.30) | 0.92 (0.28) |
| Missing observations^1^ | 0 | 11 | 0 | 6 | 0 | 32 | 0 | 2 | 0 | 56 | 0 | 75 |
| **Teenage pregnancy** | | | | | | | | | | | | |
| Yes | 0.22 (0.41) | NA | 0.29 (0.45) | NA | 0.39 (0.48) | NA | 0.55 (0.49) | NA | 0.27 (0.44) | NA | 0.38 (0.48) | NA |
| No | 0.78 (0.41) | NA | 0.70 (0.45) | NA | 0.61 (0.48) | NA | 0.45 (0.49) | NA | 0.73 (0.44) | NA | 0.64 (0.48) | NA |
| Missing observations | 0 | NA | 0 | NA | 0 | NA | 0 | NA | 0 | NA | 0 | NA |
| **FGM/C** | | | | | | | | | | | | |
| Yes | 0.61 (0.48) | 0.54 (0.49) | 0.78 (0.41) | 0.73 (0.44) | 0.64 (0.48) | 0.52 (0.49) | NA | NA | NA | NA | NA | NA |
| No | 0.21 (0.40) | 0.24 (0.42) | 0.11 (0.31) | 0.12 (0.32) | 0.309 (0.46) | 0.435 (0.49) | NA | NA | NA | NA | NA | NA |
| Don’t know | 0.18 (0.38) | 0.21 (0.41) | 0.11 (0.31) | 0.14 (0.35) | 0.002 (0.04) | 0.006 (0.07) | NA | NA | NA | NA | NA | NA |
| No answer | 0 | 0.0008 (0.02) | NA | NA | 0.048 (0.21) | 0.03 (0.17) | NA | NA | NA | NA | NA | NA |
| Missing observations | 0 | 0 | 0 | 0 | 2 | 6 | NA | NA | NA | NA | NA | NA |
| **Ever received any form of SRH information/ education** | | | | | | | | | | | | |
| Yes | 0.79 (0.40) | 0.75 (0.43) | 0.23 (0.42) | 0.25 (0.43) | 0.86 (0.34) | 0.60 (0.48) | 0.82 (0.38) | 0.76 (0.42) | 0.48 (0.49) | 0.42 (0.49) | 0.76 (0.42) | 0.33 (0.46) |
| No | 0.21 (0.40) | 0.25 (0.43) | 0.77 (0.42) | 0.75 (0.43) | 0.14 (0.34) | 0.40 (0.48) | 0.18 (0.38) | 0.24 (0.42) | 0.52 (0.49) | 0.58 (0.49) | 0.24 (0.42) | 0.67 (0.46) |
| Missing observations^2^ | 0 | 0 | 0 | 25 | 0 | 36 | 0 | 0 | 0 | 41 | 0 | 28 |
| **Parental education** | | | | | | | | | | | | |
| Mother having some level of education | | | | | | | | | | | | |
| Yes | 0.08 (0.27) | 0.13 (0.33) | 0.85 (0.36) | 0.88 (0.32) | 0.21 (0.40) | 0.18 (0.38) | 0.40 (0.49) | 0.45 (0.49) | 0.59 (0.49) | 0.43 (0.49) | 0.47 (0.49) | 0.54 (0.49) |
| No | 0.92 (0.27) | 0.87 (0.33) | 0.15 (0.36) | 0.12 (0.32) | 0.79 (0.40) | 0.82 (0.38) | 0.60 (0.49) | 0.55 (0.49) | 0.40 (0.49) | 0.57 (0.49) | 0.53 (0.49) | 0.46 (0.49) |
| Missing observations^1^ | 0 | 18 | 0 | 71 | 0 | 25 | 0 | 157 | 0 | 142 | 0 | 314 |
| Father having some level of education | | | | | | | | | | | | |
| Yes | 0.17 (0.37) | 0.24 (0.42) | 0.88 (0.32) | 0.90 (0.30) | 0.22(0.41) | 0.24 (0.42) | 0.62 (0.48) | 0.65 (0.47) | 0.47 (0.49) | 0.46 (0.49) | 0.63 (0.48) | 0.63 (0.48) |
| No | 0.83 (0.37) | 0.76 (0.42) | 0.12 (0.32) | 0.10 (0.30) | 0.78 (0.41) | 0.76 (0.42) | 0.38 (0.48) | 0.35 (0.47) | 0.53 (0.49) | 0.54 (0.49) | 0.37 (0.48) | 0.37 (0.48) |
| Missing observations^1^ | 0 | 32 | 0 | 88 | 0 | 68 | 0 | 286 | 0 | 145 | 0 | 454 |
| **Household size** | | | | | | | | | | | | |
| One to two | 0.28 (0.44) | 0.19 (0.38) | 0.05 (0.22) | 0.04 (0.21) | 0.05 (0.21) | 0.03 (0.17) | 0.09 (0.29) | 0.07 (0.25) | 0.10 (0.29) | 0.12 (0.32) | 0.03 (0.19) | 0.02 (0.15) |
| Three to four | 0.34 (0.47) | 0.27 (0.44) | 0.42 (0.49) | 0.43 (0.49) | 0.30 (0.45) | 0.19 (0.39) | 0.48 (0.50) | 0.37 (0.48) | 0.45 (0.49) | 0.43 (0.49) | 0.23 (0.42) | 0.19 (0.39) |
| Five to seven | 0.30 (0.46) | 0.43 (0.49) | 0.45 (0.49) | 0.47 (0.49) | 0.41 (0.49) | 0.43 (0.49) | 0.31 (0.46) | 0.41 (0.48) | 0.37 (0.48) | 0.35 (0.47) | 0.35 (0.47) | 0.44 (0.49) |
| Eight or more | 0.08 (0.27) | 0.11 (0.32) | 0.08 (0.26) | 0.06 (0.23) | 0.23 (0.42) | 0.35 (0.47) | 0.11 (0.31) | 0.15 (0.35) | 0.08 (0.27) | 0.10 (0.30) | 0.37 (0.48) | 0.35 (0.47) |
| Missing observations | 0 | 0 | 0 | 0 | 0 | 0 | 0 | 0 | 0 | 0 | 0 | 0 |
| **Study area** | | | | | | | | | | | | |
| Study area 1 | 0.59 (0.49) | 0.50 (0.50) | 0.46 (0.49) | 0.48 (0.49) | 0.50 (0.50) | 0.50 (0.50) | 0.52 (0.50) | 0.51 (0.50) | 0.54 (0.49) | 0.52 (0.49) | 0.25 (0.43) | 0.24 (0.43) |
| Study area 2 | 0.41 (0.49) | 0.50 (0.50) | 0.54 (0.49) | 0.52 (0.49) | 0.50 (0.50) | 0.50 (0.50) | 0.48 (0.50) | 0.49 (0.50) | 0.46 (0.49) | 0.48 (0.49) | 0.52 (0.49) | 0.51 (0.49) |
| Study area 3 | NA | NA | NA | NA | NA | NA | NA | NA | NA | NA | 0.21 (0.41) | 0.24 (0.42) |
| Missing observations | 0 | 0 | 0 | 0 | 0 | 0 | 0 | 0 | 0 | 0 | 0 | 0 |
| **Total observations** | **682** | **1,596** | **611** | **1,534** | **474** | **1,368** | **619** | **1,595** | **602** | **1,470** | **335** | **1,448** |

Data are presented in means (standard deviation). The last row presents numbers.

Data have been rounded.

The demographic data presented for women between 18-24 years represent the dataset used for the regression analysis. This data did not include missing data due to the data management process. Hence, the values are 0. Variables that were not used in the regression analysis are 'having ever dropped out of school' (except for Indonesia) and 'FGM/C'.

Missing data for 15-24 years are presented. No child marriage and teenage pregnancy data are presented for the 15-24 years age group, because of the definitions of child marriage and teenage pregnancy with ‘cut off points’ at 18 and 20 years respectively.

^1^ Included ‘don’t know’ or ‘no answer’.

^2^ Included ‘don’t know’ or ‘no answer’ except in Malawi where these answer options were not included.
